# Potential of Dry-Cured Ham Bones as a Sustainable Source to Obtain Antioxidant and DPP-IV Inhibitory Extracts

**DOI:** 10.3390/antiox12061151

**Published:** 2023-05-25

**Authors:** Gisela Carrera-Alvarado, Fidel Toldrá, Leticia Mora

**Affiliations:** Instituto de Agroquímica y Tecnología de Alimentos (CSIC), Avenue Agustín Escardino 7, Valencia, 46980 Paterna, Spain; gcarrera@iata.csic.es (G.C.-A.); ftoldra@iata.csic.es (F.T.)

**Keywords:** antioxidant peptides, dry-cured ham, DPP-IV inhibitor, enzymatic hydrolysis, meat byproducts

## Abstract

The utilization of animal bones as a protein source could be used as a sustainable pathway for the production of bioactive compounds. In this study, bones were pretreated with pepsin enzyme (PEP) and then sequentially hydrolyzed with Alcalase (PA) and Alcalase, as well as Protana prime (PAPP). The degree of hydrolysis, antioxidant activity, and DPP-IV inhibitory activity were measured. All three hydrolysates showed antioxidant and DPP-IV inhibitory activity; however, the highest result in both bioactivities was obtained with the PAPP hydrolysate. The obtained free amino acid content was 54.62, 88.12, and 668.46 mg/100 mL of hydrolyzed in PEP, PA, and PAPP, respectively. Pepsin pretreatment did not significantly affect the degree of hydrolysis; however, it is suggested that it promoted the cleavage of certain bonds for subsequent protease action. Accordingly, a total of 550 peptides were identified in PEP hydrolysate, 1087 in PA hydrolysate, and 1124 in PAPP hydrolysate using an LC-MS/MS approach. Pepsin pretreatment could be an effective method in the utilization of bone sources for the production of antioxidant and hypoglycemic peptides.

## 1. Introduction

In the context of approaching a sustainable food industry, the production of bioactive peptides from meat byproducts represents an interesting and efficient way to improve the circular economy of the sector. The obtained peptides can be used as bioactive ingredients in food, feed, pet food, and in food supplements or complement the production of functional products that can mitigate or prevent several pathophysiological conditions improving global metabolic health [1]. In this sense, peptides with multifunctional activities are of interest in the management of different diseases. For example, cardiovascular diseases and type 2 diabetes are often associated with each other and related to increased oxidative stress, which can lead to cell and tissue damage [2].

Recently, different byproducts derived from the meat industry have been described as a source of potential antioxidant or antidiabetic peptides. Thus, antioxidant peptides produced from bovine or porcine blood hydrolysates [3], fish byproducts [4], and bone or cured ham trimmings [5] have been recently described. These peptides could positively influence organism protection by counteracting oxidative stress caused by high concentrations of reactive oxygen species (ROS) as a consequence of the oxidation of membranes and lipoprotein [6], or they could also be recycled to protect meat or other foods against oxidation and bacterial growth, and thus become a natural alternative to synthetic preservatives through their use as bioactive ingredients [3,7]. Furthermore, there have been recent studies regarding DPP-IV inhibitory peptides generated from meat and fish byproduct hydrolysates, such as salmon skin or trimmings [8], chicken blood [9], or rainbow trout byproducts [10]. In this sense, several studies have suggested that the regulation of glucose levels in the human bloodstream can be improved by the consumption of dietary proteins, protein hydrolysates, peptides, and amino acids through both direct and/or indirect mechanisms [11]. The mechanism of action of DPP-IV inhibitors is based on prolonging the insulinotropic activity of incretin hormones (GLP-1 and GIP) by blocking the enzymatic activity of DPP-IV and thereby improving glycemic control [12,13].

Dry-cured ham is a product of high nutritional, economic, and cultural value and is of enormous importance for the Mediterranean meat industry; it is estimated that 70% of its total production is distributed in a sliced format [14]. Bones are one of the most abundant byproducts in pork processing, representing approximately 35% of the live weight of an adult animal [15]. It is estimated that the world meat industry produces 1.3 × 10^8^ tons of animal bone per year. Bone is an organic-inorganic compound containing between 22–45% collagen and 50–74% hydroxyapatite. Its high collagen protein content makes it an interesting option for the production of highly-valued hydrolysates [15]. 

The aim of this study was the utilization of dry-cured ham bones as a substrate to produce hydrolysates showing antioxidant and DPP-IV inhibitory activities using pretreatment with the enzyme pepsin, followed by an intense hydrolysis with the Alcalase enzyme and a combination of Alcalase and Protana prime enzymes.

## 2. Materials and Methods

### 2.1. Chemicals and Reagents

The enzymes used for hydrolysis, Alcalase and Protana prime, were purchased from Novozymes. Porcine pepsin enzyme, 1,1-diphenyl-2-picrylhydrazyl (DPPH), 2,2′-azino-bis (3-ethylbenzothiazoline-6-sulfonic acid) (ABTS), butylated hydroxytoluene (BHT), ferric chloride, (±)-6-hydroxy-2 acid, 5,7,8-tetramethylchromane-2-carboxylic acid (Trolox), potassium hexacyanoferrate (III), ascorbic acid, and the DPP-IV Inhibitor Screening Kit (MAK203) were purchased from Sigma-Aldrich Co. (St. Louis, MO, USA). All reagents were of analytical grade.

### 2.2. Sample Preparation and Enzymatic Hydrolysis

Femoral bone samples were obtained from 24-month-aged Spanish dry-cured hams obtained from a local supermarket (*n* = 5). The bones were cut into very small pieces, defatted, lyophilized, and crushed to a powder in a RETSCH MM 400 mill (Thermo Fisher Scientific Inc. Madrid, Spain). The defatting was carried out with running water at 45 °C until clean water was obtained. A pre-digestion was performed prior to enzymatic hydrolysis, with the aim of improving the subsequent action of proteases. In each replicate, 0.5 g of dry-cured ham bone powder (containing 28.8% crude protein, N × 6.25) was resuspended in 2.9 mL of 100 mM sodium acetate, pH 3.6, and pepsin at 2000 U/mL for 3 h at 37 °C. Subsequent sequential enzymatic hydrolysis was performed with 4% Alcalase (2.4 AU-A/g) for 2 h at 65 °C and pH 8, followed by 5% Protana prime (1067 LAPU/g) for 12 h at 60 °C and pH 6.8. The pH of the samples was adjusted with 2.5 M NaOH. The remaining concentration in the samples was 166.67 mg bone/mL. The hydrolysis treatments were: C = control (without hydrolysis); PEP = pepsin at 2000 U/mL; PA = pepsin at 2000 U/mL + Alcalase 2.4 L at 4%; PAPP = pepsin at 2000 U/mL + Alcalase 2.4 L at 4% + Protana prime at 5%. The enzymatic activity was stopped by heating in a water bath at 85 °C for 10 min. The samples were then kept at −20 °C until analysis. Each treatment was performed in triplicate. 

The degree of hydrolysis was evaluated according to the methodology described by Church et al. [16]. Briefly, the OPA solution was prepared by combining the following reagents: 50 mL of 100 mM di-sodium tetraborate decahydrate, 5 mL of 20% SDS (*wt*/*wt*), 80 mg of OPA dissolved in 2 mL of methanol, and 200 μL of 2-mercaptoethanol. A 100 μL sample of each hydrolysate previously diluted 100-fold in bidistilled water was used and incubated with 3.4 mL of the OPA reagent for 2 min at room temperature. The absorbance was measured at 340 nm using an ultraviolet-visible spectrophotometer (UV-Vis) (Cary 60; Agilent Technologies, Santa Clara, CA, USA). The degree of hydrolysis (DH) was calculated according to the following equation:

$$DH\;\left(\%\right)=\frac{ABS*1.934*d}{c}$$

where *ABS* is the absorbance of the samples, *d* is the dilution factor, and *c* is the protein concentration of the sample (g/L). All measurements were prepared in triplicate.

### 2.3. Antioxidant Activity

#### 2.3.1. DPPH Radical Scavenging Activity

DPPH activity was evaluated as the reduction of the absorbance of the DPPH radical in the samples relative to the absorbance of the control sample. To perform the assay, 100 µL of each hydrolysate at an initial concentration of 20 mg/mL was mixed with 500 µL of ethanol (HPLC grade) and 125 µL of DPPH reagent (0.02% in ethanol). The resulting mixture was incubated for 60 min at room temperature in the dark for subsequent absorbance detection in a UV-vis spectrophotometer (Cary 60, Agilent Technologies). Ethanol was used as a negative control and BHT (20 mg/mL) as a positive control. The activity was assayed using the following equation: DPPH inhibitory activity = (Absorbance of the negative control—Absorbance of the sample) × 100/Absorbance of the negative control [17].

#### 2.3.2. Ferric-Reducing Antioxidant Power (FRAP)

Assay was carried out by mixing 140 µL of each sample with 140 µL of 200 mM PBS at pH 6.6 and 140 µL of potassium hexacyanoferrate (III) at a concentration of 10 mg/mL. The mixture was then incubated at a temperature of 50 °C for 20 min. For the stop reaction, 200 µL of trichloroacetic acid (100 mg/mL) was added, followed by centrifugation at 200· *g* for 10 min. The resulting supernatant (400 µL) was mixed with 400 µL of bidistilled water and 80 µL of ferric chloride (1 mg/mL). This mixture was incubated in the dark for 10 min. The absorbance was measured at 700 nm using a UV-vis spectrophotometer (Cary 60, Agilent Technologies). A higher absorbance reading indicates a stronger ferric-reducing power. As a positive control, BHT (20 mg/mL) was used [18].

#### 2.3.3. ABTS

ABTS (7 mM) radical cation was dissolved in 2.45 mM potassium persulfate and was allowed to stand in the dark for 16 h to prepare the ABTS radical reagent solution. The resulting solution was diluted with 50 mM PBS (pH 7.4) to ensure an absorbance of 0.70 (±0.02) at 734 nm detected in a UV-vis spectrophotometer (Cary 60, Agilent Technologies). Then, 10 µL of the different hydrolysates at an initial concentration of 20 mg/mL were mixed with 990 µL of ABTS radical reagent solution, after 6 min of incubation at room temperature in the dark the absorbance at 734 nm was read. As a positive control, ascorbic acid was used and PBS was used as a negative control. The ABTS radical scavenging activity was expressed as nmol Trolox Equivalent Antioxidant Capacity (TEAC) per mL of hydrolysate when calibrated against the standard curve (0.05–2 mM) [19].

### 2.4. DPP-IV Inhibitory Activity

This assay is based on a high-throughput screening method for potential DPP4 inhibitors, which inhibit the degradation of glucose-dependent insulinotropic polypeptide and glucagon-like peptide 1 by the DPP-IV enzyme. DPP-IV activity is measured by cleaving the substrate to produce a fluorescent product (λex = 360/λem = 460 nm), proportional to the enzyme activity present. Inhibitory activity was assayed following the steps in the protocol supplied with the kit. Prior to the assay, each hydrolysate was diluted with bidistilled water to a concentration of 20 mg/mL. The required amount of inhibition reaction mixture was prepared, which contained 49 µL of DPP-IV assay buffer + 1 µL of DPP-IV enzyme, in addition to the enzyme reaction mixture, which contained 23 µL of DPP-IV assay buffer + 2 µL of DPP-IV substrate. The assay was performed by adding, in a 96-well plate, 50 µL of the sample, the inhibitor (Sitagliptin), and/or enzyme control (DPP-IV assay buffer), and 50 µL of the inhibition reaction mixture was mixed. The mixture was incubated at 37 °C for 10 min. After incubation, 25 µL of the enzyme reaction mixture was added and the fluorescence was measured in a multimode microplate reader (BMG LABTECH, Ortenberg, Germany) in kinetic mode for 30 min at 37 °C. The relative inhibition rate was calculated according to the following equation:

$$\%\;Relative\;Inhibition=\frac{Slope_{EC}-Slope_{M}}{Slope_{EC}}*100$$

where: *Slope_SM_* = the slope of the sample inhibitor, and *Slope_EC_* = the slope of the enzyme control.

### 2.5. Characterization of the Peptide Fraction

#### 2.5.1. Peptide Separation by Reversed-Phase Chromatography (RP-HPLC)

Peptide separation by RP-HPLC was performed using a Symmetry C18 column (4.6 × 250 mm, 5 μm, Waters Co., Milford, MA, USA). Phase A was 0.1% TFA bidistilled water, and phase B was 0.085% TFA in ACN:H_2_O (60:40, *v*/*v*). A 100 μL sample of each hydrolysate (100 mg/mL) was injected. The elution was monitored at 214 nm, with a flow rate of 1 mL/min in a step gradient mode, in which phase B remained at 0% for 2 min, and then linearly increased to 50% from minute 2 to 50.

#### 2.5.2. Peptide Separation by Hydrophilic Interaction Liquid Chromatography (HILIC)

Peptide separation by HILIC was performed using a ZIC^®^-pHILIC column (4.6 × 250 mm, 5 μm, Supelco Inc. Darmstadt, Germany). ACN/NH_4_Ac 5 mM (80:20, *v*/*v*) pH 6.8 with an elution gradient of 90% to 40% ACN in 20 min was used as the mobile phase. An amount of 100 μL of each hydrolysate was injected at a concentration of 100 mg/mL. The elution was monitored at 214 nm with a flow rate of 1 mL/min.

#### 2.5.3. Identification of Peptides by Mass Spectrometry in Tandem

The Ekspert 425 liquid chromatography system (Eksigent, Redwood City, CA, USA) was used to inject 3 µL of the sample, which was then processed through a C18-CL trap column (3 µ 120 Ᾰ, 350 μm × 0.5 mm; Eksigent) and desalted with 0.1% TFA for 3 min at 5 µL/min. The peptides were subsequently loaded onto a C18-CL analytical column (3 µ 120 Ᾰ, 0.075 × 150 mm; Eksigent) and equilibrated in 5% ACN and 0.1% FA (formic acid). Elution was performed with a linear gradient from 5 to 40% B in A for 20 min (A: 0.1% FA; B: ACN, 0.1% FA) at a 300 nL/min flow rate. Peptides were analyzed using a nanoESI qQTOF 6600 plus TripleTOF mass spectrometer (SCIEX, Framinghan, MA, USA), where peptides were ionized in a Nano Optiflow by applying 3.0 kV to the spray emitter at 200 °C. The analysis was performed in a data-dependent mode with MS1 scans of 350–1400 *m/z* obtained for 250 ms and MS2 experiments acquired at 100–1500 *m/z* for 25 ms in high sensitivity mode. The criteria used for switching were: 1+ to 5+ charge, minimum intensity, 100 cps. Up to 100 ions were selected for fragmentation after each survey scan. Dynamic exclusion was set to 15 s.

Spectra were analyzed using ProteinPilot v 5.0 (SCIEX, Framingham, MA, USA). The peak list was generated from the 6600 plus TripleTOF wiff files. The Paragon algorithm (ProteinPilot v 5.0) was used to search the SwissProt and Uniprot Cordata database with the following parameters: no enzyme specificity, IAM cys-alkylation, no taxonomy restriction, and the search effort was set to all. Protein clustering was performed using the Pro group algorithm. Protein cluster formation in Pro group is only fully guided by observed peptides. Since the observed peptides are determined from experimentally acquired spectra, clustering can be considered to be guided by the use of spectra. Then, unobserved regions of the protein sequence play no role in explaining the data. A trypsin K562 (500 ng) digestion was also analyzed to ensure system sensitivity. Under these conditions, 1819 proteins (FDR < 1%) were identified in a 20 min gradient.

#### 2.5.4. Matrix-Assisted Laser Desorption/Ionization Time-of-Flight Mass Spectrometry (MALDI-ToF MS) Analysis

An amount of 2 μL of the sample was diluted 1:50 in the matrix (10 mg/mL of α-Cyano-4-hydroxycinnamic acid (CHCA from Bruker, GM2520) in ACN: 0.1% TFA (70:30, *v*/*v*)); 1 μL of this solution was spotted onto the MALDI plate and allowed to air-dry at room temperature. The resulting mixtures were analyzed in a tims TOF flex (Bruker) in MALDI operation, in reflector positive mode at 700–3500 *m*/*z* and with a laser intensity of 60%. The core facility provides the spectrum in .xy format and .emf file. The xy files were analyzed using the tool mMass (http://www.mmass.org/, accessed on 7 December 2022).

#### 2.5.5. Free Amino Acids (FAAs)

The concentration of free amino acids was measured using an Agilent HPLC system with a Pico Tag^®^ C18 3.9 × 300 mm column (Waters Corp., Milford, MA, USA). Deproteinization was performed by homogenizing 300 μL of hydrolysates of Pep, PA, PAPP, and C (control) with 0.01 N HCl (1:4; *w*/*v*) for 5 min and then centrifuged at 10,000× *g* for 20 min at 4 °C. Samples were derivatized using the methodology described by Aristoy and Toldrá [20], using 5 mM norleucine as the internal standard. Chromatographic separation was performed according to the methodology described by Flores et al. [21]. Briefly, 70 mM sodium acetate with 2.5% ACN, pH 6.55, was used as phase A and as phase B; ACN: H_2_O: MeOH was used in a ratio of 45:40:15. The elution was monitored at 254 nm, with a temperature of 52 °C and a flow rate of 1 mL/min. For the quantification of free amino acids, the response factors previously calculated for each amino acid in a mixed standard series were considered. The results were reported as mg FAAs/100 mL of hydrolysate.

### 2.6. Statistical Analysis

The results of the degree of hydrolysis, antioxidant and DPP-IV inhibitory activities, and free amino acids were analyzed by one-way ANOVA (*p* < 0.05), using an LSD test for the comparison of means. Statistical analysis was performed with R software v. 4.2.0 (R Foundation for Statistical Computing, Vienna, Austria). All data were reported as the mean of three replicates and error bars were derived from the standard deviations of the data.

## 3. Results and Discussion

### 3.1. Degree of Hydrolysis (DH)

**Figure 1:**
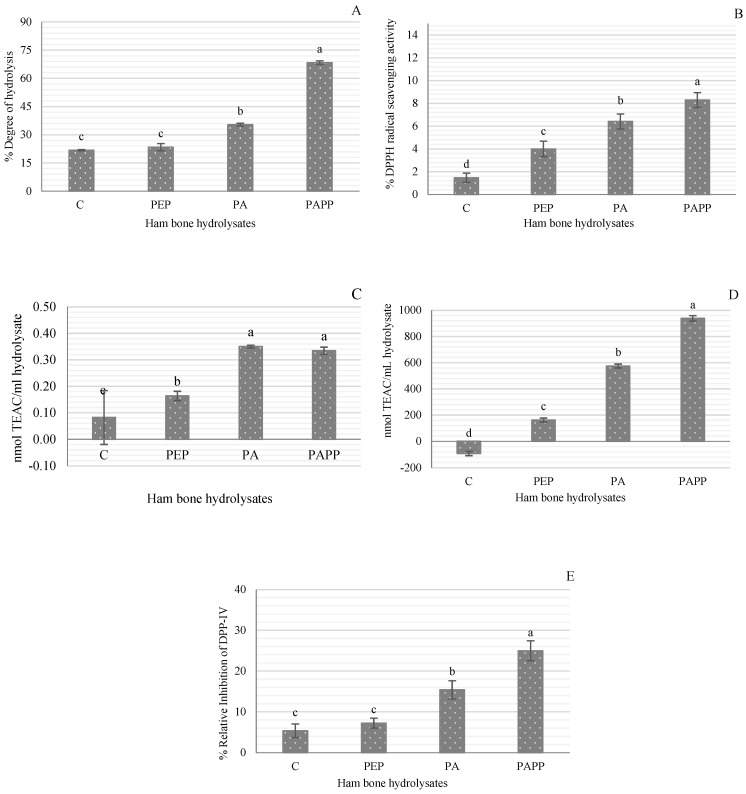
(**A**) Degree of hydrolysis, (**B**) DPPH radical scavenging activity, (**C**) Ferric-reducing antioxidant power, (**D**) ABTS inhibition activity, and (**E**) DPP-IV inhibition activity of the different dry-cured ham bone hydrolysates. C = control (without hydrolysis); PEP = Pepsin at 2000 U/mL (3 h, 37 °C and pH 3.6); PA = Pepsin at 2000 U/mL (37 °C and pH 3.6, 3 h) + Alcalase 2.4 L at 4% (65 °C, pH 8, 2 h); PAPP = Pepsin at 2000 U/mL (37 °C and pH 3.6, 3 h) + Alcalase 2.4 L at 4% (65 °C, pH 8, 2 h) + Protana prime at 5% (60 °C, pH 6.8, 12 h). Data represent mean ± SD (*n*  =  3). Different lowercase letters (a–d) indicate statistically significant differences between hydrolysates (*p* < 0.05).

The sequential hydrolysis using Alcalase and Alcalase and Protana prime enzymes after the pretreatment with pepsin (PA and PAPP, respectively) significantly increased the degree of hydrolysis of ham bones, reaching a DH of 68.38% in the PAPP hydrolysate. The PA hydrolysate reached a DH of 35.36%. The sample pretreated with pepsin showed a DH of 23.41% and non-significant differences (*p* > 0.05) from the control sample (Figure 1A). In a previous study regarding pork bone hydrolysates, the highest DH achieved was 12.14% using the Neutrase^®^ enzyme (2.5%) for 2 h at 55 °C and pH 7.0 [22]. In a recent study with bovine bones pretreated with lipase enzyme at 0.08% (4 h at 40 °C and pH 7.5), a DH of 12.58% was reported [23]. The higher DH values obtained in this study in comparison with previous studies can be attributed to (i) dry-cured ham bones being involved in a proteolytic process where a large number of peptides and free amino acids are generated [14,24], and (ii) pepsin pretreatment could promote enzyme accessibility to target sites as a consequence of collagen denaturation. Yao et al. [23] suggested that enzymatic pretreatment plays a crucial role in the efficiency of enzymatic hydrolysis, since the unique structure of bone, surrounded by adipose tissue and the insoluble characteristic of proteins, makes the entry of proteases into the cleavage sites difficult, resulting in a low yield of peptide products and negatively affects the comprehensive utilization of bone resources. 

### 3.2. Antioxidant Activity

DPPH radical scavenging activity, ferric-reducing antioxidant power, and ABTS radical scavenging assay are methods that are generally classified as one-electron-transfer-based methods, in which antioxidants reduce substrates by providing an electron to radicals and oxides [25]. Due to the diverse nature of antioxidant compounds, three different assays were used to analyze the antioxidant activity of dry-cured ham bone hydrolysates. Figure 1B–D shows the highest antioxidant activity in the PAPP hydrolysate (8.28% DPPH radical scavenging, 0.1462 absorbance in FRAP, and 93.80 nmol TEAC/mL). PA hydrolysate also showed good antioxidant activity; even the FRAP result (0.1493) was statistically similar (*p* > 0.05) to that of PAPP hydrolysate. In all three assays, the highest antioxidant activity was found in the hydrolysates with the highest degree of hydrolysis (PAPP and PA). Although there was no significant difference in DH between the PEP hydrolysate and the control sample, their antioxidant activity was statistically different. These results indicate that the antioxidant power of dry-cured ham bone hydrolysates depends on the type and size of peptides generated during hydrolysis. It has been reported that the potential of a peptide to act as an antioxidant is mainly determined by its molecular weight, amino acid content, and its specific position within the peptide sequence [26,27]. In particular, peptides with small molecular sizes increase efficiency and lead to easier access to the oxidant/antioxidant system [28]. This is because small peptides, unlike large ones, have a greater ability to donate electrons to free radicals, thus disrupting the oxidative chain by more efficiently scavenging superoxide anion, hydroxyl radical, and hydrogen peroxide [4,29]. Najafian and Babji [30] stated that most antioxidant peptides extracted from food sources show molecular weights ranging from 500 to 1800 Da. As an example, the molecular weight of antioxidant peptides in silver carp and Skipjack tuna byproduct hydrolysates was lower than 1 kDa [4,28].

The control sample also showed certain antioxidant activity in the DPPH and FRAP assays. This result can be attributed to the antioxidant properties reported in Spanish dry-cured ham [31,32,33].

### 3.3. DPP-IV Inhibitory Activity

Figure 1E shows the DPP-IV enzyme inhibitory activity of the different hydrolysates. Samples C and PEP had the lowest DPP-IV inhibition (<7.5%) and were statistically equal (*p* > 0.05), so the pepsin pretreatment did not significantly influence the production of DPP-IV inhibitory peptides. The sample sequentially hydrolyzed with Alcalase and Protana prime (PAPP) also resulted in higher DPP-IV inhibitory activity (24.99%). As for the control inhibitor, sitagliptin, the percentage of inhibition was 98.80%. The type of protease used is of great importance in the generation of DPP-IV inhibitory peptides. In a previous study with chicken blood hydrolysates, the combination of Alcalase (2%, 2 h at 55 °C) and Protana prime (5%, 16 h at 55 °C) also generated DPP-IV inhibitory peptides, with an inhibition rate of 60.55% [9]. Cheung and Li-Chan [34] noted that consecutive hydrolysis using two enzymes is effective to produce multifunctional hydrolysates, as occurs in steelhead gelatin hydrolysates treated with papain (4%, 2 h at 65 °C and pH 6) followed by Corolase^®^N (1%, 2 h at 55 °C and pH 7.5) with in vitro DPP-IV and ACE inhibitory activity.

### 3.4. Characterization of the Generated Peptides Profile by HPLC and MALDI-MS Analysis

**Figure 2:**
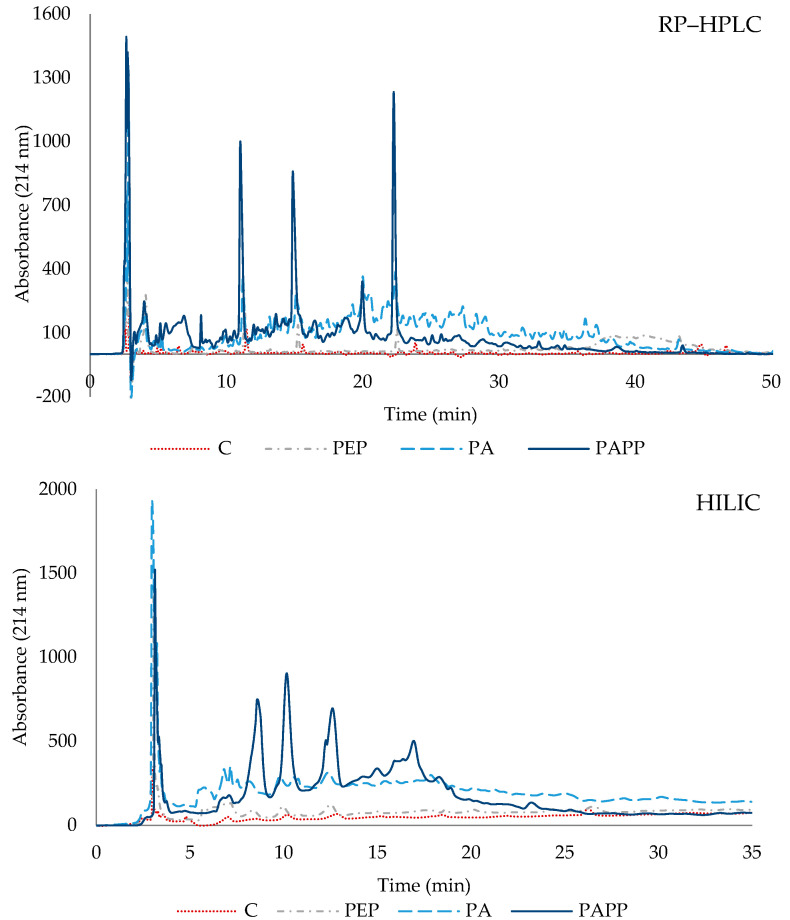
Elution profile by RP–HPLC and HILIC chromatography of the hydrolysates PEP (Pepsin at 2000 U/mL); PA (Pepsin at 2000 U/mL + 4% Alcalase 2.4 L); PAPP (Pepsin at 2000 U/mL + 4% Alcalase 2.4 L + 5% Protana Prime); and the control sample (C).

Peptide elution profiling of the hydrolysates was performed by reverse-phase high-performance liquid chromatography (RP-HPLC) and hydrophilic interaction chromatography (HILIC). Both methodologies allow the peptide obtained to be analyzed according to their hydrophobicity, which is highly related to their amino acid composition. Figure 2 shows the chromatographic profiles of each sample. Using both methodologies, the peptide profile of the control differs from the hydrolyzed samples (PEP, PA, and PAPP). Higher absorbance is observed in the PAPP sample. This indicated that sequential hydrolysis with Alcalase and Protana prime results in a more intense hydrolysis process. Regarding this, Alcalase has been described to be very active by degrading hydrophobic amino acid peptide bonds [30].

**Figure 3:**
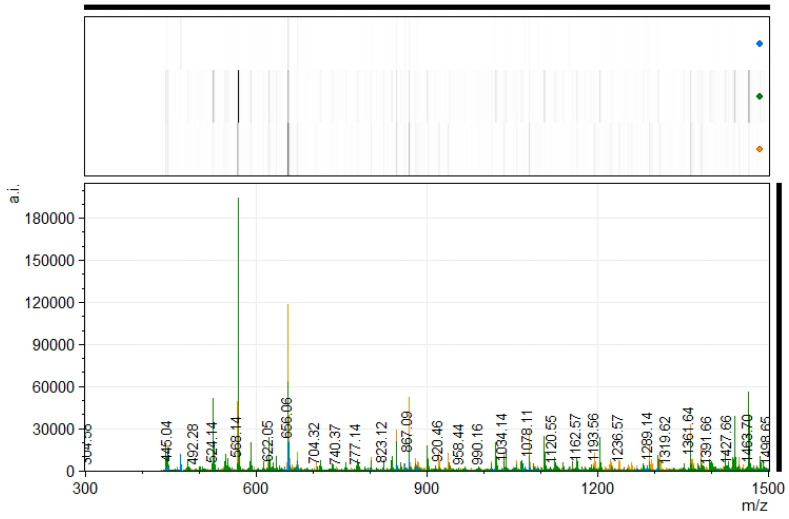
MALDI-TOF mass spectrum of the hydrolysates PEP (Pepsin at 2000 U/mL); PA (Pepsin at 2000 U/mL + 4% Alcalase 2.4 L), and PAPP (Pepsin at 2000 U/mL + 4% Alcalase 2.4 L + 5% Protana Prime). Blue color corresponds to P hydrolysate, green to PA, and yellow to PAPP.

Dry-cured ham bone protein hydrolysates were also analyzed using MALDI-TOF mass spectrometry (Figure 3). The results obtained showed a mass-to-charge (*m*/*z*) profile of peptides between 400 and 1500 *m*/*z*. The PA sample showed many peptides, the intensity of which decreased in the case of PAPP, probably because some of the peptides disappeared as the degree of hydrolysis increased. The MALDI profile of the P sample proved the hydrolysis occurred during the pretreatment, which was very useful for the subsequent action of the studied proteases.

### 3.5. Identification of the Generated Peptides Using Mass Spectrometry in Tandem (LC-MS/MS) 

In addition to these results, a mass spectrometry approach was used to identify the peptides generated after pepsin pretreatment and enzymatic hydrolysis with Alcalase and Protana prime. Table 1 shows the total number of unique peptide sequences identified in each hydrolysate. A total amount of 25 peptide sequences in the control sample changed to 550, 1087, and 1124 in PEP, PA, and PAPP samples, increasing the number of peptides with the hydrolysis intensity. Regarding this, pepsin is a gastrointestinal proteolytic enzyme responsible for degrading dietary proteins into small peptides and amino acids to be subsequently absorbed and used in the synthesis of new proteins and energy [35]. On the other hand, when hydrolyzing with Alcalase, the cleavage sites are different and the proteins or protein fragments are further broken down due to the extensive hydrolysis it provides. 

These results are associated with the biological activities of the hydrolysates, as an increasing number of peptides is also associated with a smaller size of these peptides as they are generated from the same proteins of origin. Thus, DPP-IV and antioxidant activities are often reported to be associated with low molecular weight peptides. 

**Figure 4:**
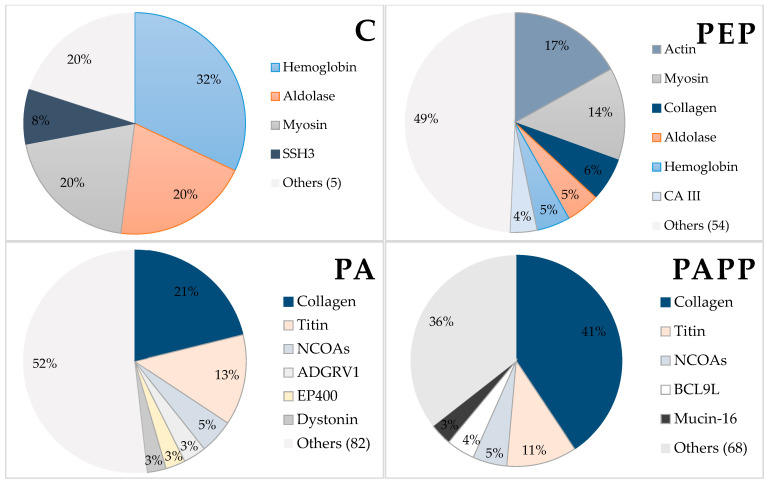
Protein distribution according to the number of unique peptides identified in each type of sample. Unique peptides have been used in the distribution analysis. C = control (without hydrolysis); Pep = Pepsin at 2000 U/mL (3 h, 37 °C and pH 3.6); PA = Pepsin at 2000 U/mL (37 °C and pH 3.6, 3 h) + Alcalase 2.4 L at 4% (65 °C, pH 8, 2 h); PAPP = Pepsin at 2000 U/mL (37 °C and pH 3.6, 3 h) + Alcalase 2.4 L at 4% (65 °C, pH 8, 2 h) + Protana prime at 5% (60 °C, pH 6.8, 12 h).

The peptides identified have been grouped according to their protein of origin using ProteinPilot™ v 5.0 (SCIEX, Framingham, MA, USA). Thus, Figure 4 shows the distribution of proteins according to the number of peptides identified in each sample. Accordingly, the control sample shows that most of the peptides identified came from the proteins hemoglobin, aldolase, myosin, and SSH3. After pepsin digestion, peptide fragments of actin, myosin, collagen, and proteins were in the majority, whereas the distribution of hemoglobin and aldolase decreased. In the PA sample, the highest percentage of peptides originated from collagen (21%) and titin (13%). These proteins also showed the highest number of sequences in the PAPP sample, together accounting for 52% of the peptides identified. Tarnutzer et al. [36] stated that the most abundant proteins in mammals are albumin, hemoglobin, histones, actin, serpin, and then collagen, the latter representing about 25 to 35% of bone proteins. Collagen, when enzymatically hydrolyzed, produces a large number of peptides that can be bioactive. In this regard, Gallego et al. [5] reported antioxidant activity in cured ham bone broths and attributed such bioactivity to collagen-derived peptides generated after cooking.

### 3.6. Free Amino Acids

Proteins are polymers of amino acids linked by a specific type of covalent bond. However, when they go through a proteolysis process, proteins can degrade, generating peptides and free amino acids, which are related to the nutritional and functional characteristics of the final product [37]. Table 2 shows the free amino acid content of each hydrolysate (PEP, PA, PAPP) and the control sample. Regarding the control sample, pretreatment with pepsin doubled the FAAs content, and hydrolyzing with Alcalase (PA) tripled this content. While, upon sequential hydrolysis with Alcalase + Protana prime (PAPP), the total FAAs increased up to 25-fold. These results may be related to the degree of hydrolysis achieved and the type of protease used in the hydrolysis. The high degree of hydrolysis in the PAPP hydrolysate suggests the production of peptides with lower molecular weight than the PA hydrolysate. Alcalase has endo-protease activity with broad specificity and naturally cleaves internal peptide bonds, resulting in proteins mainly composed of medium-sized peptides [27]. Whereas Protana prime is an exo-peptidase that releases small peptides and free amino acids from proteins.

Based on the total amount of free amino acids, the PEP, PA, and PAPP hydrolysates were characterized by having a high amount of essential amino acids (~45%); while the content in the control sample was up to 20 points lower. As for hydrophobic amino acids, differences in relative abundances were also observed. In the control sample (C), 19.88% of the free amino acid content is hydrophobic amino acids (including Ala, Pro, Val, Leu, Ile, Met Phe, Tyr, and Trp), in PEP it is 33.56%, in PA it is 35.46%, and in PAPP it is 38.13%. Research has demonstrated that amino acids with hydrophobic character in peptide sequences can interfere with oxidative chain reactions by strongly donating electrons. This ability can help peptides across the cell membrane and eliminate intracellular ROS [30]. It has also been described that the activity of DPP-IV inhibitory peptides is largely influenced by their amino acid composition and they are generally rich in hydrophobic amino acids, as these enhance the interaction with the active site of the DPP-IV enzyme [13]. Similarly, Arg and Tyr residues have been reported to exhibit DPP-IV inhibitory activity [2].

## 4. Conclusions

The application of pepsin pretreatment before enzymatic hydrolysis is an effective method for the production of bioactive compounds from bone sources. This represents an improvement in the utilization of meat industry byproducts in a sustainable manner. The results obtained show that peptide extracts of ham bone hydrolysates have antioxidant and DPP-IV inhibitory activity. These extracts could be used as bioactive ingredients in functional foods.

## Figures and Tables

**Table 1:** Number of peptides identified in each dry-cured ham hydrolysate after LC-MS/MS analysis.

Sample	Num. of Unique Peptides
C	25
PEP	550
PA	1087
PAPP	1124

**Table 2:** Free amino acid composition (mg/100 mg of hydrolysate) of cured ham bone hydrolysates.

Free Amino Acids	Sample			
	C	PEP	PA	PAPP
Aspartic acid (Asp)	5.74 ± 0.09 ^c^	3.73 ± 0.00 ^d^	9.96 ± 0.22 ^b^	38.14 ± 0.18 ^a^
Glutamic acid (Glu)	3.49 ± 0.11 ^d^	4.74 ± 0.06 ^c^	7.57 ± 0.34 ^b^	54.67 ± 0.42 ^a^
Serine (Ser)	2.95 ± 0.07 ^d^	5.3 ± 0.03 ^c^	6.7 ± 0.09 ^b^	34.14 ± 0.28 ^a^
Asparagine (Asn)				23.37 ± 0.59 ^a^
Glycine (Gly)	2.31 ± 0.11 ^c^	3.86 ± 0.17 ^b c^	4.91 ± 0.19 ^b^	47.76 ± 1.66 ^a^
Glutamine (Gln)				26.73 ± 0.81 ^a^
Histidine (His)			1.82 ± 0.16 ^b^	9.43 ± 0.33 ^a^
Threonine (Thr)	1.68 ± 0.14 ^d^	3.58 ± 0.09 ^c^	5.63 ± 0.27 ^b^	30.11 ± 0.81 ^a^
Alanine (Ala)	1.64 ± 0.11 ^d^	3.61 ± 0.30 ^c^	5.67 ± 0.56 ^b^	54.06 ± 0.7 ^a^
Arginine (Arg)	1.67 ± 0.05 ^d^	5.3 ± 0.36 ^c^	7.76 ± 0.77 ^b^	57.21 ± 0.78 ^a^
Proline (Pro)				19.56 ± 0.65 ^a^
Tyrosine (Tyr)	1.88 ± 0.15 ^c^	3.68 ± 0.09 ^b^	4.62 ± 0.08 ^b^	24.95 ± 1.22 ^a^
Valine (Val)		2.34 ± 0.17 ^b^	4.14 ± 0.13 ^b^	37.54 ± 1.86 ^a^
Methionine (Met)			2.84 ± 0.48 ^b^	13.31 ± 0.52 ^a^
Isoleucine (Ile)		1.45 ± 0.10 ^c^	2.57 ± 0.12 ^b^	23.53 ± 0.54 ^a^
Leucine (Leu)	1.83 ± 0.16 ^d^	4.66 ± 0.05 ^c^	7.29 ± 0.21 ^b^	50.44 ± 0.55 ^a^
Phenylalanine (Phe)		2.58 ± 0.06 ^c^	4.11 ± 0.49 ^b^	24.51 ± 0.28 ^a^
Tryptophan (Trp)				6.97 ± 0.1 ^a^
Lysine (Lys)	3.74 ± 0.07 ^d^	9.79 ± 0.07 ^c^	12.53 ± 0.88 ^b^	92.06 ± 0.64 ^a^
Total (mg/100 mL hydrolysate)	26.37 ± 0.45 ^d^	54.62 ± 0.07 ^c^	88.12 ± 1.36 ^b^	668.46 ± 5.64 ^a^

PEP (Pepsin at 2000 U/mL); PA (Pepsin at 2000 U/mL + 4% Alcalase 2.4 L); PAPP (Pepsin at 2000 U/mL + 4% Alcalase 2.4 L + 5% Protana Prime); and the control sample (C). Values shown are the mean ± standard deviation of three replicates. Means in the same row without a common letter in superscript significantly differ (*p* < 0.05).

## Data Availability

Data will be available upon request to authors.
